# Aqueous Extracts of *Morus alba* Root Bark and *Cornus officinalis* Fruit Protect against Osteoarthritis Symptoms in Testosterone-Deficient and Osteoarthritis-Induced Rats

**DOI:** 10.3390/pharmaceutics12121245

**Published:** 2020-12-21

**Authors:** Sunmin Park, Bo Reum Moon, Ji Eun Kim, Hyun Joo Kim, Ting Zhang

**Affiliations:** 1 Department Food & Nutrition, Obesity/Diabetes Center, Hoseo University, Asan 31499, Korea; cherish_moon@naver.com (B.R.M.); tjtnwls56@hanmail.net (J.E.K.); qlalzl777@naver.com (H.J.K.); 20195723@vision.hoseo.edu (T.Z.); 2 Department of Bio-Convergence System, Hoseo University, Asan 31499, Korea

**Keywords:** bone mineral density, lean body mass, articular cartilage, inflammation, pain behavior, testosterone deficiency

## Abstract

Water extracts of both *Morus alba* L. root bark (MBW) and *Cornus officinalis* Siebold and Zucc fruit (CFW) have traditionally been used to promote men’s health in the elderly in Asia. We determined that the 12-week consumption of MBW and CFW could alleviate testosterone-deficiency syndrome and osteoarthritis (OA) symptoms in testosterone-deficient rats, and the action mechanisms were explored. Rats with bilateral orchiectomy (ORX) were fed a 45% fat diet containing either 0.5% MBW (ORX-MBW), 0.5% CFW(ORX-CFW), or 0.5% dextrin (ORX-CON). Sham-operated rats also received 0.5% dextrin (Non-ORX-CON). After 8 weeks of treatment, all rats had an injection of monoiodoacetate (MIA) into the left knee, and they continued the same diet for the additional 4 weeks. ORX-CFW and ORX-MBW partially prevented the reduction of serum testosterone concentrations and decreased insulin resistance, compared to the ORX-CON. ORX-CFW and ORX-MBW protected against the reduction of bone mineral density (BMD) and lean body mass (LBM) compared to the ORX-CON. The limping and edema scores were lower in the order of the ORX-CON, ORX-CRF = ORX-MBW, and Non-ORX-CON (*p* < 0.05). The scores for pain behaviors, measured by weight-distribution on the OA leg and maximum running velocity on a treadmill, significantly decreased in the same order as limping scores. ORX-MBW protected against the increased expression of matrix metalloproteinase (MMP)-3 and MMP-13 and reduced the production of inflammatory markers such as TNF-α and IL-1β, by MIA in the articular cartilage, compared to the ORX-CON (*p* < 0.05). The cartilage damage near the tidemark of the knee and proteoglycan loss was significantly less in ORX-MBW than ORX-CON. In conclusion, MBW, possibly CFW, could be effective alternative therapeutic agents for preventing osteoarthritis in testosterone-deficient elderly men.

## 1. Introduction

Osteoarthritis is a degenerative joint disease, and its prevalence is influenced by gender in persons over age 50. After menopause, women have increased osteoarthritis risk. However, only a few studies have investigated the association between testosterone deficiency and osteoarthritis risk in elderly men, and those results have been inconsistent [1,2]. However, serum testosterone concentrations are positively associated with cartilage volume, but the loss of cartilage volume for 2 years does not induce osteoarthritis [2]. In a prospective cohort study, osteoarthritis risk is associated with lower serum androstenedione concentrations in obese and overweight men [1]. Therefore, lowering circulating serum concentrations of sex hormones may influence osteoarthritis etiology in men. Like bone mass, cartilage volume is greater in men, and the incidence of osteoarthritis does not appear to be associated with serum testosterone. However, a longer duration of low testosterone concentrations due to increased life span may show its association with osteoarthritis. 

Low circulating testosterone concentration is relatively frequent in elderly men, and they often suffer from testosterone-deficiency syndrome (TDS). However, testosterone replacement therapy is uncommon since its risks vs. benefits have been debated [3]. Oral administration of natural testosterone does not maintain proper levels of testosterone due to its quick degradation in the liver. Long-term acting intramuscular injection of testosterone ester has been used for treating hypogonadism. However, it has disadvantages, including muscular injections every 1–3 weeks and fluctuations in serum testosterone concentrations [3]. However, in most cases, testosterone replacement therapy has potential adverse effects, including prostate cancer, breast cancer, and prostate nodule and induration, and severe lower urinary tract symptoms. Therefore, testosterone replacement therapy is not commonly used, and alternative therapies, such as herbal medicines, are needed. 

*Morus alba* L. (white mulberry) root bark and *Cornus officinalis* Siebold and Zucc fruit (Japanese Cornelian Cherry) have been traditionally used for treating various metabolic diseases, including diabetes and inflammatory diseases in elderly Asians [4,5]. *Morus alba* L. root bark has been used for treating inflammation in the lungs with coughing [4,5]. *Morus alba* L. root bark has been reported to have hypoglycemic and diuretic activities, to improve immune response, and to have antiviral, anti-inflammatory, and antiallergic activities [6,7]. It contains α-amyrin and β-amyrin (triterpenoids), β-sitosterol (steroid), morusin, cyclomorucin, and kuwanon (flavonoids), and 1-deoxynojirimycin and fagomine (alkaloids) [8,9]. In Asia, *Cornus officinalis* Siebold and Zucc fruit is known to be good for men’s health to promote sperm function and bone marrow and to reduce urinary tract problems [4,5]. *Cornus officinalis* Siebold and Zucc fruit is also known to have antioxidant, anti-inflammatory, antidiabetic, and neuroprotective activities [10,11,12]. It contains morroniside and loganin (iridoids), quercetin and kaempferol derivatives (flavonoids), pelargonidin 3-*O*-galactoside, cyanidin 3-*O*-galactoside, and delphinidin 3-*O*-galactoside (anthocyanins), gallic acid and malate (organic acids), and terpenoids (oleanolic acid) [13]. Therefore, *Morus alba* L. root bark and *Cornus officinalis* Siebold and Zucc fruit reduce inflammation and oxidative stress. Moreover, in our preliminary studies, *Morus alba* L. root bark and *Cornus officinalis* Siebold and Zucc fruit effectively inhibited aromatase in MCF-7 cells, suggesting that they promote testosterone production. In the present study, we hypothesized that the consumption of *Morus alba* L. root bark and fruits of *Cornus officinalis* Siebold and Zucc would alleviate the symptoms of testosterone-deficiency and osteoarthritis in a testosterone-deficient animal model. We examined the hypothesis in bilateral orchidectomized (ORX) and monoiodoacetate (MIA)-injected rats fed a high-fat diet.

## 2. Materials and Methods

### 2.1. Phenolic and Flavonoid Contents and Index Compounds

The root bark of *Morus alba* L. and fruit of *Cornus officinalis* Siebold and Zucc purchased from the Kyung Dong Herbal Market (Seoul, Korea) were validated for authenticity by Dr. Young Seng Joo (Woo-Suk University, Jeonju, Korea). Ground herbs were extracted for 3 h in 90 °C distilled water, filtered through filter paper (No. 2), and concentrated using a rotary evaporator and lyophilization. The yields of the *Morus alba* L. root bark and *Cornus officinalis* fruit aqueous extracts were 17.5% and 19.6%, respectively. Total phenolic compounds in each powder were measured by Folin–Ciocalteu reagent and expressed as mg gallic acid equivalents·g^−1^ [14]. Each extract was mixed with Folin–Ciocalteu reagent (10:1, *v*/*v*; Sigma, St Loise, MO, USA), and after 3 min, 10% (*w*/*v*) Na_2_CO_3_ was added to each reaction mixture and held in the dark for 60 min. The absorbance of each reaction mixture was determined at 725 nm wavelength using a UV spectrophotometer (Perkin Elmer, Boston, MA, USA). Total flavonoids were determined using a modified method published by Davis [15] with quercetin as the standard. Each extract was mixed with 5% sodium nitrite, and after 5 min, 10% aluminum chloride (3:1:1, *v*/*v*/*v*; Sigma) was added. Each mixture was incubated at room temperature for 6 min. It was neutralized with 1 N NaOH. Its absorbance was measured at 510 nm using a UV spectrophotometer (Perkin Elmer). 

All HPLC analyses were performed on an Agilent 1100 series HPLC instrument (Agilent Technologies, Santa Clara, CA, USA) equipped with an autosampler (G1313A), column oven (G1316A), binary pump (G1312), DAD detector (G1315B), and degasser (G1379A). The contents of the indicated compounds in *Cornus officinalis* fruits (gallic acid, morroniside, and loganin) and *Morus alba* L. root bark (kuwanon G and morusin) were analyzed with an Optimapak C18 column (4.6 × 250 mm, 5 µm, Waters Co., Milford, MA, USA). The mobile phase consisted of the solvents acetonitrile, methanol, and distilled water with 0.1% acetic acid (10:5:85) for index compounds for the *Cornus officinalis* fruit extracts. The mobile phase was made of water (A) and methanol (B) and the following gradients were used: 0 min, A:B 85:15 (*v*/*v*) for 5 min; A:B 30:70 (*v*:*v*) for 25 min and A:B 0:100 (*v*:*v*) for 10 min. The flow rate of the mobile phase was 0.5 mL/min for *Cornus officinalis* fruit and 1 mL/min for *Morus alba* L. root bark, and the column temperature was 20 °C for *Cornus officinalis* fruit and 25 °C for *Morus alba* L. root bark. UV detection was at 250 nm for *Cornus officinalis* fruit and 270 nm for *Morus alba* L. root bark. The injection volume was 10 μL. The contents of the indicated compounds were quantified using standards (1–500 ug/mL) of kuwanon G (Ensol Bioscience, Daejeon, Korea), morusin, gallic acid, morroniside, and loganin (Sigma-Aldrich, St. Loise, MO, USA). 

### 2.2. Bilateral Orchiectomies and Experimental Design

Forty male Sprague–Dawley rats weighing 195 ± 14 g (average 7 weeks old) from DBL (Eum Sung, Korea) were separately accommodated in stainless steel cages in the animal facility with a controlled environment (23 °C and with a 12/12 h light/dark cycle). All animal research protocols have followed the guidelines for the National Institutes of Health guide for the care and use of laboratory animals (NIH Publications No. 8023, revised 1978) and were approved by the Animal Care and Use Review Committee of Hoseo University (Asan, Korea; HSIACUC-14-04).

**Figure 1:**
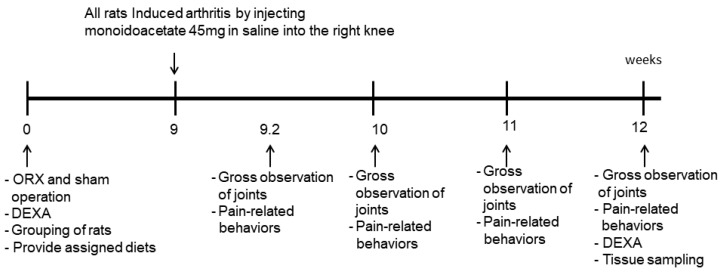
Timeline of the experiments. ORX-CON, ORX male rats with induced osteoarthritis and 43% fat diet with 0.5% dextrin; ORX-CFW, ORX male rats with induced osteoarthritis and 43% fat diet plus 0.5% CFW; ORX-MBW: ORX male rats with induced osteoarthritis and 43% fat diet plus 0.5% MBW; Non-ORX-CON, Sham-operation of ORX male rats with induced osteoarthritis and 43% fat diet with 0.5% dextrin. CFW, Water extract of *Cornus officinalis* Siebold and Zucc fruits; MWB, water extract of *Morus alba* L root bark; DEXA, dual-energy X-ray absorptiometry.

The experimental time schedules are shown in Figure 1. There was a marked increase in testicular weight in male rats on the 33rd day. Male rats have secondary sexual maturity at 41 and 54 days [16]. Thirty rats aged 8 weeks were subjected to bilateral orchiectomies after being anesthetized using intraperitoneal injections of ketamine and xylazine (10 and 1 mg/kg BW) after acclimation for 1 week. A mid-ventral incision was made in the testicles, including the part of the spermatic cord, were excised, and the incision was sutured. After 1 day, the ORX rats fed their assigned diets. Age-matched male rats had a sham-ORX operation (Non-ORX) by rubbing the testicle instead of its removal. After the 9th week from the surgery, all ORX and Non-ORX rats were given intra-articular injections of MIA (4 mg/50 μL saline; Sigma) through the patellar ligament of the left knee, using a 26-gauge needle after anesthetizing with intramuscular injections of a ketamine and xylazine mixture (100 and 10 mg, respectively) [17].

### 2.3. Diets and Experimental Design

High-fat semipurified diets based on the AIN-93 formulation were provided to exacerbate the TDS and OA [18]. Each diet had the following micronutrient content: carbohydrate 40% energy (En%); protein 20 En%; fat 40 En%. Either 0.5% aqueous extracts of *Morus alba* root bark (MBW), 0.5% aqueous extracts of *Cornus officinalis* fruits (CFW), or 0.5% dextrin for controls were added into the high-fat diets. Starch and sugar (carbohydrate), casein (protein), and lard (fat) were the major sources of macronutrients (CJ Co., Seoul, Korea). Water and the respective diets were freely accessible for the 8-week study. 

The ORX rats induced osteoarthritis were randomly and blindly divided into three treatment groups (n = 10 for each group) as follows: (1) ORX-MBW group provided the diet containing MBW powder, (2) ORX-CFW group given the diet containing CFW powder, and (3) ORX-CON fed the diet containing dextrin. The Non-ORX-CON group consisted of 10 sham-operated rats fed the same diet as ORX-CON.

At the beginning of the 8th week, all rats were fasted overnight and subjected to oral glucose tolerance tests (OGTT) by orally administering glucose (2 g glucose/kg BW) as previously described [19]. At 3 days after OGTT, an intraperitoneal insulin tolerance test (IPITT) was conducted by injecting 0.75 U insulin per kg body weight after withholding food for 6 h. Serum glucose and insulin levels were analyzed with a Glucose Analyzer II (Beckman-Coulter, Palo Alto, CA, USA) and rat ultrasensitive insulin kit (Crystal Chem, Elk Grove Village, IL, USA) with an ELISA reader, respectively. The homeostasis model assessment estimate of insulin resistance (HOMA-IR; fasting insulin (µIU/mL) × fasting glucose (mM)/22.5) was to estimate the degree of insulin resistance.

### 2.4. MIA-Induced Osteoarthritis Animal Model and Osteoarthritis Progression 

At 3, 7, 14, and 21 days after MIA injection, each rat was weighed and carefully inspected to assess knee joint swelling and gait disturbances under natural conditions in the cages for gross observation, where they were allowed to move freely. Swelling and limping were classified as no change (0), mild (1), moderate (2), and severe (3) based on the severity of the symptoms [20]. The two trained inspectors who conducted all assessments were blinded to treatment details throughout the study, and the average values from two independent inspectors were used for the statistical analysis. Each Tuesday at 10 AM, bodyweight was recorded after 16 h fasting.

### 2.5. Pain-Related Behavior Assessments 

The Incapacitance test was used to evaluated pain-related behavior using a hind paw limb weight-bearing apparatus (Linton Incapacitance Tester, UK) and the maximum running speed on a treadmill as previously described. These tests indicate joint discomfort and may be useful for the discovery of novel pharmacologic agents for treating human osteoarthritis [21]. Animals were acclimated for 30 min before the Incapacitance test. Measurements were performed five times in each rat, and the average of the middle three values was used to calculate the percent weight distribution of the left hind paw as previously described [17]. 

The maximal running speed on a treadmill is considered as a diagnostic criterion for osteoarthritis. Rats were acclimated to treadmill running at 40 cm/s for 1 min and then increased to 50 cm/s for 1 min. Subsequently, the treadmill velocity increased by 5 cm/s at 1 m intervals until running could not be maintained, and the rats slid into the back of the treadmill. The maximum running speed was defined as the fastest speed that the rats could maintain for 20 s. 

The locomotive activity was determined using a Linton AM1053 Activity Monitor, which uses a three-dimensional array of infrared beams placed around clear Perspex cages with AmLogger software (Linton Instruments, UK). The rats were allowed to adapt to the cages for 30 m, and then the movement was monitored for 1 h during the dark phase of the light/dark cycle when the rats were most active.

### 2.6. Body Composition

After the 12-week treatment period, the rats were anesthetized, and body composition, including bone mineral density (BMD), lean body mass (LBM), and fat mass was measured by dual-energy X-ray absorptiometry (DEXA; Norland pDEXA Sabre; Norland Medical Systems Inc., Fort Atkinson, WI, USA). Rats were then sacrificed, and blood collected for serum by cardiocentesis. Epididymal fat was surgically removed and weighed, and it was considered as the visceral fat mass. 

Serum testosterone levels were measured by ELISA (Enzo Life Sciences, Farmingdale, NY, USA). Lipid profiles in the circulation were measured using colorimetry kits from Asan Pharmaceutical (Seoul, Korea).

### 2.7. Isolation of Total RNA from Articular Cartilage and Real-Time PCR 

Articular cartilage samples from five rats of each group were collected at 21 days after MIA injection. Each cartilage sample was separately powdered with a cold steel mortar and pestle and then mixed with a monophasic solution of phenol and guanidine isothiocyanate (TRIzol reagent, Life Technologies, Rockville, MD, USA) for total RNA extraction according to the manufacturer’s instructions. The cDNA was synthesized from 1 μg RNA extracted from each rat using a superscript III reverse transcriptase kit (Life Science Technology). Each cDNA transcript was prepared using the SYBR Green mix (Bio-Rad, Richmond, CA, USA) for realtime PCR as previously described [17,20]. Inflammatory and cartilage degradation related genes assessed included tumor necrosis factor (TNF)-α, interleukin (IL)-1β, matrix metalloproteinase (MMP)-3, and MMP-13 genes as described previously [17]. The gene expression levels in unknown samples were quantitated using the comparative cycles of the threshold method [17]. 

### 2.8. Histopathological Analysis of Knee Joints 

Rats were sacrificed at 21 days post-MIA injection, and knee articular bones and joints were histologically examined for narrowing and loss of joint region, cartilage erosion, and osteophyte formation [17,20]. Five micrometers of knee joints were embedded in paraffin and stained with hematoxylin and eosin (H-E) and Safranin-O fast green. Histopathological changes were quantitatively expressed by the following scoring system [20]. The depth of cartilage damage was scored on a scale of 0–5 where 0 was normal; 1 minimal (affecting only the superficial zone); 2 mild (the upper-middle zone invaded); 3 moderate (substantial invasion into the middle zone); 4 major (marked invasion into the deep zone but not to the tidemark); 5 severe (full-thickness degradation into the tidemark). The extents of tibial plateau involvement and proteoglycan loss were scored as 1 (minimal), 2 (mild), 3 (moderate), and 4 (severe). The two trained inspectors who scored the joints were blind to the treatments, and average values from the two independent inspectors were used for the statistical analysis.

### 2.9. Statistical Analysis 

All data were statistically evaluated using SAS software version 7 (SAS Institute, Cary, NC, USA). All results were expressed as means ± standard deviations. One-way ANOVA was used to evaluate the significance of differences in the metabolic effects of ORX-CON, ORX-MBW, ORX-CFW, and Non-ORX-CON at a single time point at the end of the experiment. Significant differences in the main effects among the groups were identified by Tukey’s test at *p* < 0.05. 

## 3. Results

### 3.1. Polyphenol and Flavonoid Contents of CFW and MBW

MBW contained 18.2 and 2.3 mg total polyphenols and flavonoids per g extract, respectively, whereas CFW had 42.7 and 14.2 mg per g extract (Table 1). The HPLC chromatograms of standards and index compounds of CFW and MBW were given in Figure S1. Index compounds in CFW were gallic acid, morroniside, and loganin, at 0.45 ± 0.03, 11.0 ± 0.09, 6.2 ± 0.04 μg/g extract, respectively (Table 1). MBW contained 2.2 ± 0.1 kuwanon G and 1.6 ± 0.1 morusin μg/g extract (Table 1).

### 3.2. Global Observations of Osteoarthritis Symptoms

**Figure 2:**
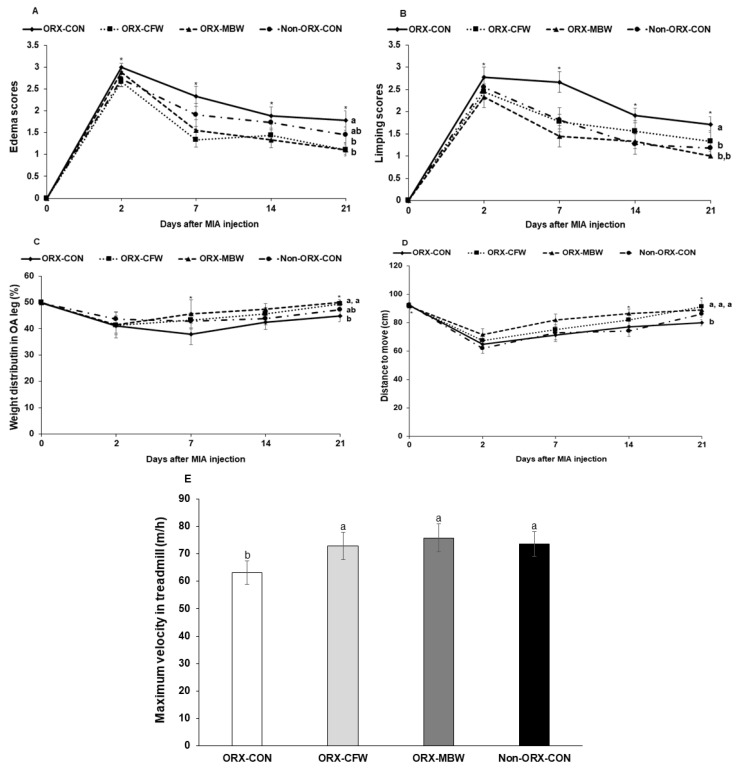
Gross observation of osteoarthritis symptoms and pain-related behaviors at 3, 7, 14, and 21 days after an intra-articular injection of monoiodoacetate (MIA). ORX-CON, orchidectomized (ORX) male rats with induced osteoarthritis were fed with 43% fat diet with 0.5% dextrin; ORX-CFW, ORX male rats with induced osteoarthritis were fed 43% fat diet with 0.5% CFW; ORX-MBW: ORX male rats with induced osteoarthritis with 43% fat diet with 0.5% MBW; Non-ORX-CON, Sham-ORX operated male rats with induced osteoarthritis fed a 43% fat diet with 0.5% dextrin. CFW, water extract of *Cornus officinalis* Siebold and Zucc fruits; MWB, water extract of *Morus alba* L. root bark (**A**) edema scores, (**B**) limping scores, (**C**) weight distribution between MIA- and saline-injected legs, and (**D**) distance to move during locomotive activity for 20 min at 2, 7, 14, and 21 days after MIA injection. (**E**) The maximum velocity on a treadmill on the 21st day after MIA injection. Each data point and error bar represent the mean *±* SD from 10 rats per group. * Significant treatment effect among the groups by one-way ANOVA test at *p* < 0.05. ^a,b^ Different letters indicate significant differences among the treatment groups at the 21st day as identified by Tukey’s test at *p* < 0.05. The ‘a’ letter on the group indicated a significant difference of mean value from the ‘b’ letter on the group at *p* < 0.05.

After intra-articular MIA injection in the left joint, osteoarthritis symptoms and pain-related behaviors occurred regardless of ORX (Figure 2). The symptoms of osteoarthritis such as limping, swelling, and weight distributions between legs significantly increased in ORX-CON compared to the Non-ORX-CON. ORX-CFW and ORX-MBW alleviated osteoarthritis symptoms, compared to ORX-CON (Figure 2). The MIA injected left knee was beginning to show edema and limping at day 2 after the injection in all MIA-injected rats (Figure 2A). Non-ORX-CON exhibited lower edema scores than ORX-CON, but it was not significantly different on day 21 (*p* = 0.08). However, ORX-CFW and ORX-MBW showed less edema than ORX-CON on days 7, 14, and 21. By day 14, the edema alleviation in ORX-CFW and ORX-MBW was greater than in the Non-ORX-CON (Figure 2A). ORX-CON rats exhibited more limping at days 2, 7, 14, and 21 than the Non-ORX-CON rats. Limping was not improved until day 7 in ORX-CON, but after day 7 it improved (Figure 2B). ORX-CFW and ORX-MBW markedly ameliorated limping until day 7, and then it stayed the same. However, limping status steadily improved in Non-ORX-CON (Figure 2B); but limping was not completely normalized in any of the groups.

### 3.3. Pain-Related Behavior Tests for Osteoarthritis

Pain-related behavior was measured by the asymmetric weight distribution, locomotive activity, and maximum velocity of running on a treadmill. In MIA-injected rats, weight distribution was not evenly distributed between the legs. Without pain, the right and left legs had equal weight distributions, but pain decreased the weight distribution on the affected leg to less than 50%. The weight distribution of the left leg was 43.5% on day 2 due to the pain and stayed at a similar level until day 21 (Figure 2C). Weight-distributions between both legs were improved in the ORX-CFW and MBW groups compared to the ORX-CON, but they were not different between ORX-CON and Non-ORX-CON (*p* = 0.09). The unequal weight distributions were significantly alleviated in ORX-MBW compared to the other groups, and they were improved in ORX-MBW and ORX-CFW compared to the ORX-CON at 21 days (Figure 2C).

Locomotive activity monitoring revealed that all rats had less movement until day 2 after MIA injection due to pain, and then the movement increased in all groups (Figure 2D). Locomotive activity did not differ between ORX-CON and Non-ORX-CON until day 14, but it increased in the Non-ORX-CON on day 21 compared to the ORX-CON (Figure 2D). The locomotive activity was higher in ORX-MBW than ORX-CON on days 2, 7, 14, and 21 (Figure 2D). Furthermore, the maximum velocity on the treadmill was higher in the ORX-MBW (74.8 m/h) than the ORX-CON (63.1 m/h) on day 21 (Figure 2E). It was not significantly different between ORX-CON, ORX-CFW, and Non-ORX-CON on day 21 (Figure 2E). Therefore, pain-related behaviors were partially improved more in Non-ORX-CON and ORX-CFW than in the ORX-CON, but ORX-MBW alleviated pain-related behaviors the most.

### 3.4. The mRNA Expressions of Cytokines in Articular Cartilage of the OA Knee

**Figure 3:**
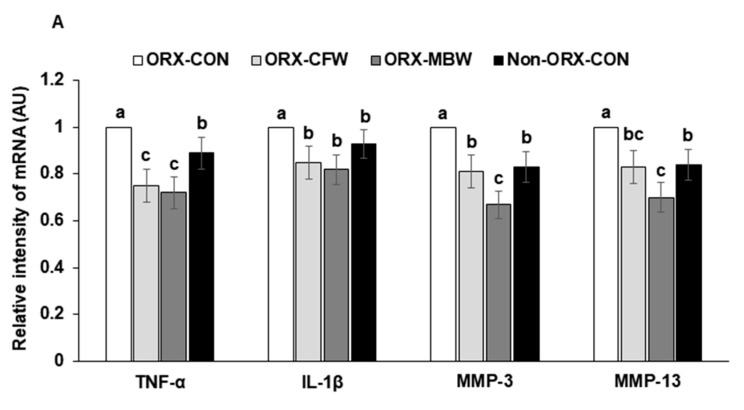

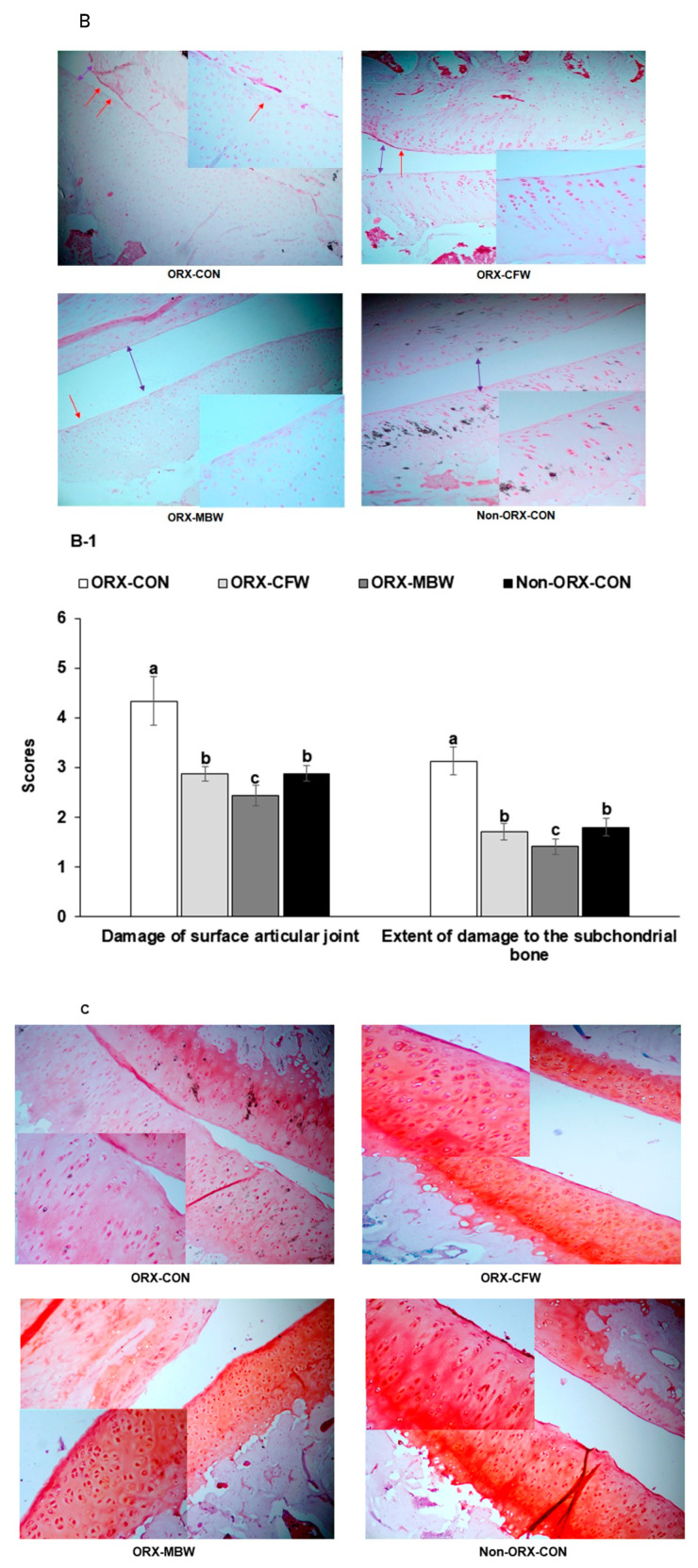

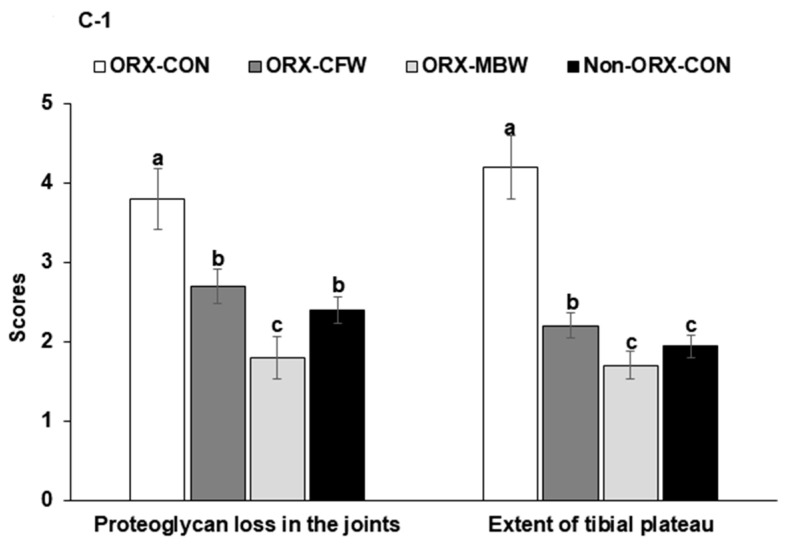
The mRNA expression of matrix metalloproteinases (MMP) and proinflammatory cytokines in the articular cartilage and the histopathological features of osteoarthritic lesions at 21 days after an intra-articular injection of monoiodoacetate (MIA). ORX-CON, orchidectomized (ORX) male rats were fed with a 43% fat diet with 0.5% dextrin; ORX-CFW, ORX male rats were fed with a 43% fat diet with 0.5% CFW; ORX-MBW: ORX male rats were fed with a 43% fat diet with 0.5% MBW; Non-ORX-CON, Sham male rats were fed with a 43% fat diet with 0.5% dextrin. CFW, water extract of *Cornus officinalis* Siebold and Zucc fruits; MWB, water extract of *Morus alba* L. root bark (**A**) mRNA expression of matrix metalloproteinase (MMP)-3, MMP-13, tumor necrosis factor (TNF)-α, and interleukin (IL)-1β in the articular cartilage. (**B**) The damage to articular cartilage damage and subchondral bone in hematoxylin-eosin (H-E) stain (magnifying power ×100 and ×200 in the image). Red and violet arrows indicated the damage to the articular cartilage and gap between the joints. (**B-1**) Scores of the damage to articular cartilage damage and subchondral bone from H-E staining. (**C**) The proteoglycan loss in the joint and extent of the tibial plateau in Safranin O fast green (SG) stain (magnifying power ×100). Red color represents the proteoglycan contents in the articular cartilage, and the violet arrow indicated the gap between joints. (**C-1**) Scores of the proteoglycan loss in the joint and extent of the tibial plateau from the SG stain. Each data point and error bar represent the mean *±* SD from five rats per group. ^a,b,c^ Different letters indicate significant differences in the treatment groups by Tukey’s test at *p* < 0.05. The ‘a’ letter on the group had a significant difference of mean value to the ‘b’ letter on the group at *p* < 0.05.

The mRNA expressions of TNF-α and IL-1β, inflammation mediators, were elevated in the articular cartilage of the MIA-injected joint in ORX-rats compared to the Non-ORX-CONs (Figure 3A). The mRNA expression of TNF-α was lower in the ORX-CFW and ORX-MBW than the Non-ORX-CON (Figure 3A). IL-1β expression was similar among the ORX-CFW, ORX-MBW, and Non-ORX-CON.

The mRNA expressions of matrix metalloproteinase (MMP)-3 and MMP-13, enzymes involved in collagen degradation, decreased in the articular cartilage of MIA-injected knees of ORX rats compared to the Non-ORX-CON (Figure 3A). ORX-CFW decreased MMP-3 and MMP-13 mRNA expression to as much as the Non-ORX-CON (Figure 3A). However, ORX-MBW lowered their mRNA expression than Non-ORX-CON. These results indicated that testosterone-deficiency itself increased proinflammatory cytokines and collagen degradation enzymes (MMP-3 and MMP-13) in the articular cartilage while their elevation was prevented in ORX-CFW and MBW. ORX-MBW protected against collagen degradation compared to the ORX-CFW.

### 3.5. Histopathological Analysis

Histological evaluations of H-E stained sections revealed that MIA injection damaged the articular cartilage and subchondral bone of the knee in ORX-CON rats (Figure 3B,B-1). All rats injected with MIA experienced impaired articular cartilage, which induced the degeneration of columnar orientation and tide mark and the penetration of subchondral bones. ORX-CON had higher histological scores of the knees compared to the Non-ORX-CON (Figure 3B,B-1). Surfaces of articular joints were degenerated, and cytokines penetrated the subchondral bone in ORX-CON more than the Non-ORX-CON, but ORX-CFW and ORX-MBW protected the joints with damage similar to the Non-ORX-CON (Figure 3B,B-1). The gap between the joints was much narrower in the ORX-CON than in the other groups. ORX-MBW showed better protection of the damage than ORX-CFW. In Safranin-O fast green staining, proteoglycan losses (reduced red staining) were shown in ORX-CON rats compared to the Non-ORX-CON and ORX-MBW protected against the loss as much as the Non-ORX-CON (Figure 3C,C-1). However, its improvement by ORX-CFW was less than that by ORX-MBW (Figure 3C,C-1). These results indicate that CFW and MBW treatment prevented the breakdown of the articular surface in ORX rats with the MIA injection, and ORX-MBW had better efficacy than ORX-CFW.

### 3.6. Energy Metabolism and Body Composition

Bodyweight at the 8th week was lower in ORX-CON than Non-ORX-CON, and it did not alter in ORX-CFW and ORX-MBW, compared to the ORX-CON (Table 2). After MIA-induced osteoarthritis, bodyweight decreased in all groups, and there was no significant difference in body weight loss among the groups. As a result, body weight at the 11th week was higher in Non-ORX-CON than ORX-CON, ORX-CFW, and ORX-MBW. ORX-CFW and ORX-MBW did not differ from the ORX-CON in body weight at the 11th week. Rats in all groups lost weight due to pain from OA during weeks 8–11, and the weight loss was higher in the order of the Non-ORX-CON, ORX-CON, ORX-MBW, and ORX-CFW (Table 2). However, food intake was not significantly different among the groups (Table 2). This indicated that ORX decreased food efficiency, and CFW and MBW did not prevent the decline. Interestingly, epididymal fat mass was not significantly different between ORX-CON and Non-ORX-CON, although body weight was much lower in ORX-CON than the Non-ORX-CON. However, ORX-MBW decreased epididymal fat mass compared to the ORX-CON (Table 2).

**Figure 4:**
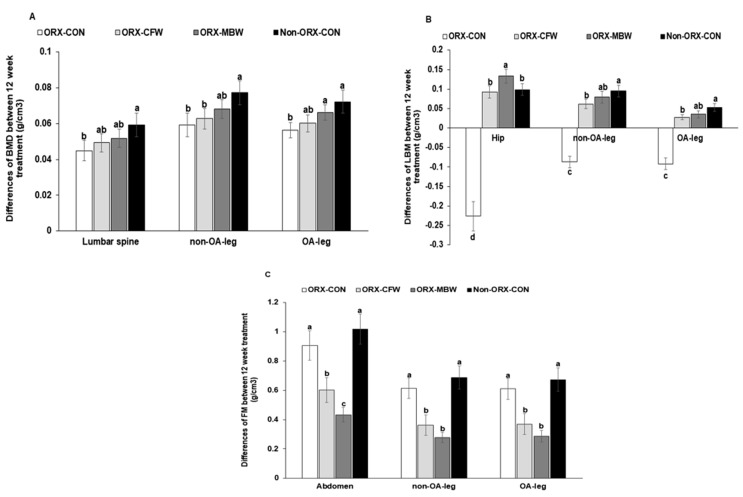
Body composition of femur and knee with intra-articular injection of monoiodoacetate (MIA) at day 0 and 21 after an intra-articular injection of monoiodoacetate. ORX-CON, orchidectomized (ORX) male rats with induced osteoarthritis and fed with 43% fat diet with 0.5% dextrin; ORX-CFW, ORX male rats with induced osteoarthritis and fed with 43% fat diet with 0.5% CFW; ORX-MBW, ORX male rats with induced osteoarthritis and fed with 43% fat diet with 0.5% MBW; Non-ORX-CON, Sham-ORX operated male rats with induced osteoarthritis with 43% fat diet with 0.5% dextrin. CFW, water extract of *Cornus officinalis* Siebold and Zucc fruits; MWB, water extract of *Morus alba* L. root bark. The red color area indicated the collagen deposition. (**A**) The difference in bone mineral density (BMD) in the lumbar spine, non-osteoarthritis (OA)-leg, and OA-leg between day 0 and 21. (**B**) The difference in lean mass in the hip, non-osteoarthritis (OA)-leg, and OA-leg between day 0 and 21. (**C**) The difference of fat mass in the abdomen, Non-OA-leg, and OA-leg between day 0 and 21. Each data point and error bar represents the mean *±* SD from 10 rats per group. ^a,b,c,d^ Different letters indicate significant differences in the treatment groups by Tukey’s test at *p* < 0.05. The ‘a’ letter on the group had a significant difference of mean value to the ‘b’ letter in the group at *p* < 0.05.

The differences in BMD between weeks 0 and 12 of rats with treatments in the lumbar spine, and between Non-OA-leg and OA-legs were much lower in the ORX-CON than the Non-ORX-CON, and they were partially prevented in the ORX-MBW, especially the OA-leg (Figure 4A). However, CFW did not have any effects on BMD. LBM in the hip, Non-OA-leg, and OA-leg decreased in ORX-CON after the 12-week experimental period, but it increased in the Non-ORX-CON (Figure 4B). The differences in LBM in the Non-OA-leg and OA-leg increased in ORX-CFW and ORX-MBW whereas the increase was higher in ORX-MBW than ORX-CFW and the increment in ORX-MBW was similar to the Non-ORX-CON (Figure 4B). Fat mass was not significantly different between ORX-CON and Non-ORX-CON, and MBW decreased fat mass the most among all groups (Figure 4C).

### 3.7. Glucose Metabolism

Serum glucose concentrations were not significantly different between ORX-CON and Non-ORX-CON, but ORX-MBW lowered serum glucose concentrations to less than ORX-CON (Table 2). Serum insulin concentrations tended to be higher in ORX-CON than the Non-ORX-CON, and they were lower in ORX-CFW and ORX-MBW than ORX-CON. Consistent with the serum glucose and insulin concentrations, HOMA-IR, an index of insulin resistance, was higher in ORX-CON than Non-ORX-CON, whereas ORX-CFW and ORX-MBW reduced HOMA-IR compared to the ORX-CON (Table 2). ORX-MBW lowered HOMA-IR more than Non-ORX-CON. Serum testosterone concentrations in the ORX-CON were about 50% of the Non-ORX-CON, and ORX-CFW and ORX-MBW decreased them compared to ORX-CON (Table 2).

After oral administration of 2 g glucose/kg body weight, serum glucose concentrations were elevated until 20–30 min, and after that, they decreased in all rats (Supplemental Figure S2A). The peak of serum glucose concentrations was highest in ORX-CON among the groups, and serum glucose levels decreased faster in Non-ORX-CON than ORX-CON. ORX-CFW and ORX-MBW decreased serum glucose concentrations faster than the ORX-CON, and ORX-MBW showed a similar decrease to the Non-ORX-CON (Supplemental Figure S2A). AUC of serum glucose at the 1st (0–30 min) and 2nd part (30–120 min) during OGTT was higher in the ORX-CON than the Non-ORX-CON (Supplemental Figure S2B). ORX-CFW and ORX-MBW lowered the AUC in the 2nd part. This indicated that ORX caused the deterioration of glucose tolerance by increasing insulin resistance, and ORX-MBW and ORX-CFW protected against the disturbance of glucose tolerance.

Serum glucose concentrations at the 6 h fasting state were higher in the ORX-CON than the Non-ORX-CON, and ORX-MBW decreased the concentrations similar to the Non-ORX-CON (Supplemental Figure S3A). After intraperitoneally injecting insulin in 6 h fasted rats, serum glucose concentrations decreased until 45 min, and then they were maintained or slightly rebounded until 90 min in all rats (Supplemental Figure S3A). ORX-CON exhibited higher serum glucose concentrations more than the Non-ORX-CON during 90 min, and ORX-CFW and ORX-MBW had similar AUC of serum glucose concentrations compared to the Non-ORX-CON (Supplemental Figure S3B).

## 4. Discussion

The incidence of osteoarthritis increases with aging and appears to be associated with age-related declines in sex hormones, including estrogen and testosterone [22]. After menopause, women are reported to have incremental increases in osteoarthritis risk compared with men of the same age [23]. This is associated with different decreasing patterns of sex hormones and body composition. Cartilage volume is correlated with LBM and sex hormones [1]. Since men have higher LBM and slower decrease of serum testosterone concentrations, the osteoarthritis risk is not elevated as quickly as in women as they age [24]. Although elderly men have a lower risk of osteoarthritis than elderly women, they are still susceptible to osteoarthritis due to decreased serum testosterone concentrations [25]. However, hormonal replacement therapy in both genders has insufficient evidence to support its safety and efficacy as an osteoarthritis intervention [26]. Alternative herbal therapy may be useful for the chronic treatment of osteoarthritis without serious adverse effects in the elderly [20]. Therefore, we examined the efficacy and mechanism of CFW and MBW for the alleviation of osteoarthritis symptoms and testosterone-deficiency syndrome in ORX rats. We demonstrated that CFW and MBW can be potential therapeutic agents for preventing testosterone-deficiency syndrome and osteoarthritis risk and that they suppress inflammation in the knee joint, which protects against the decrease in BMD, LBM, and insulin sensitivity in a testosterone-deficient animal model.

Testosterone deficiency leads to decreased LBM, increased abdominal fat mass, and ultimately increased insulin resistance in males [27]. Additionally, β-cells have androgen receptors, and testosterone potentiates glucose-stimulated insulin secretion, and it has similar insulinotropic action as glucagon-like peptide-1 [27]. Testosterone deficiency impairs glucose metabolism to increase type 2 diabetes risk in elderly men. Hyperglycemia increases insulin resistance and elevates advanced glycated endproducts (AGEs) that increase oxidative stress and the release of proinflammatory cytokines that exacerbate osteoarthritis symptoms [28]. Insulin resistance is linked to the elevation of the systemic low-grade inflammatory state in the joints [28]. In diabetic patients with osteoarthritis, diabetic treatment partially improves osteoarthritis symptoms by decreasing AGEs and insulin resistance [28]. However, the impairment of glucose metabolism may not be related to osteoarthritis. In men aged 45–65 years, there is an association between fasting glucose concentrations and only OA in the hands [29]. The present study also showed that testosterone-deficient rats had impaired glucose metabolism and insulin resistance that might exacerbate osteoarthritis symptoms. CFW and MBW improved glucose tolerance and insulin resistance while simultaneously ameliorating osteoarthritis symptoms. Therefore, testosterone deficiency and glucose metabolism are associated with osteoarthritis progression.

Osteoarthritis is a degenerative joint disease that is characterized by progressive degradation of articular cartilage [30]. It is involved in inflammation leading to irreversible structural damage in the articular cartilage, and it induces pain and stiffness and impedes movement. MIA injection into the articular cartilage of rodents does not exhibit the same etiology as human osteoarthritis. However, the MIA-injected animals have similar characteristics as osteoarthritis, including pain and damage of articular cartilage by inflammation as commonly occurs in human osteoarthritis [30]. Osteoarthritis has been studied in menopausal women, but it has not been studied much in testosterone-deficient men since the prevalence of osteoarthritis is much higher in women than men. However, testosterone deficiency is also associated with osteoarthritis risk. Castrated male rhesus macaques exhibit severe joint osteoarthritis [31]. Furthermore, higher serum total testosterone concentrations are associated with less pain in elderly men and women with severe knee osteoarthritis [22]. Higher serum testosterone concentrations are linked to a higher level of LBM, including knee cartilage volume, which delays the damage to articular cartilage. As life expectancy increases, men are increasingly susceptible to osteoarthritis, although they have higher articular cartilage volume than women [22].

MBW is known to possess hypoglycemic activity and decrease lipid peroxidation in streptozotocin-induced diabetic rats [32]. It is also reported to facilitate weight loss by suppressing appetite and improving dyslipidemia [33,34]. Oral intake of a mixture of *Acacia catechu* water extract and *Morus alba* root bark ethanol extract (50 mg/kg BW) has anti-inflammatory (TNF-α and IL-1β), collagenase (MMP-13) inhibitory, and articular cartilage protective activities in MIA-induced arthritis [35,36]. Furthermore, moracin, a flavonoid from *Mores alba* root bark protects against IL-1β-induced osteoarthritis by reducing inflammatory factors (TNF-α, IL-6, and COX2) via attenuating the NF-κB pathway [37]. Consistent with previous studies [35,36,37], the present study also demonstrated that MBW attenuated pain and proinflammatory activities in the articular cartilage along with preventing the reduction of serum testosterone concentrations in the testosterone-deficient rats with MIA-induced osteoarthritis. MBW inhibited the BMD loss only in the leg with osteoarthritis, and it was associated with the suppression of osteoarthritis progression. MBW also protected against the loss of LBM in the ORX rats by increasing the production of testosterone in the adrenal gland despite no testis. Furthermore, MBW inhibited short-term memory impairment by ORX. Thus, *Morus alba* root bark might promote the 17β-hydroxysteroid dehydrogenase in the adrenal glands to increase serum testosterone to higher concentrations than ORX-CON.

Previous scientific studies have reported that CFW has antitumor, anti-inflammatory, antidiabetic, analgesic, antioxidant, neuroprotective, antiaging, and immunoregulatory activities [13]. Traditionally, they have been known to be good for men’s health in Korea. The predominant active components of CFW are organic acids and iridoids, including morroniside, and loganin [38]. They have antioxidant and anti-inflammatory activities, and loganin modulates the serotonergic nervous system and GABAergic neurons to exert sedative and hypnotic activities [39]. It was involved in reducing osteoarthritis pain by CFW in the present study. However, CFW has not been previously studied for protection against osteoarthritis induced by MIA in an animal model. However, it has been shown to reduce inflammatory activity [40]. Along with MBW, CFW also promoted the secretion of testosterone in ORX rats. This suggested that MBW and CFW elevated 17β-hydroxysteroid dehydrogenase in the adrenal glands. Therefore, MBW and CFW can be alternative medicines for osteoarthritis in elderly men with low testosterone secretion.

Infiltration of inflammatory cells, which consist mainly of macrophages, but also T and B cells, mast cells, and natural killer cells, induces the remarkable hyperplasia of synovial cells in the early OA stage [38]. OA interventions target the suppression of inflammatory processes to reduce proinflammatory cytokines (TNF-α, IL-1, IL-6, IL-15, IL-10) in synovial membranes and articular cartilage [41]. The present study also showed that ORX-CON increased the mRNA expression of TNF-α and IL-1β, proinflammatory cytokines, compared to Non-ORX-CON, and ORX-CFW and ORX-MBW reduced their mRNA expressions more than the ORX-CON and even Non-ORX-CON. Furthermore, low-grade inflammation and innate immune activation with immune dysfunction develop as a result of aging, smoking, and physical inactivity [42]. Chronic low-grade inflammation with obesity and metabolic syndrome and innate immune activation, like cytokine storms, trigger progressive degeneration in OA joints [43]. The increase in insulin resistance and systemic inflammation might exacerbate the inflammation in the joints, OA pathology. ORX decreased skeletal muscle mass and induced insulin resistance, and it also elevated serum TNF-α concentrations, an indicator of systemic inflammation, in the present study. Moreover, ORX markedly suppressed serum testosterone concentrations. Low concentrations of sex hormones are associated with chronic musculoskeletal pain in the elderly, but it is related to women more than men [44]. Low plasma androstenedione concentration is also reported to be associated with an increased risk of OA in the knee and hip of overweight and obese men [1]. Consistent with the previous studies [45,46], both CWF and MBW prevented the increase in insulin resistance and inflammation that are linked to alleviating OA symptoms. Therefore, ORX exacerbates OA symptoms, but CFW and MBW prevented the exacerbation by reducing insulin resistance and systemic and joint inflammation and by elevating serum testosterone concentrations.

## 5. Conclusions

The present study suggested that testosterone deficiency decreased LBM and increased inflammation and abdominal fat mass, which exacerbated osteoarthritis symptoms. Water extracts of *Morus alba* L. root bark and *Cornus officinalis* Siebold and Zucc fruits protected against testosterone deficiency-induced osteoarthritis symptoms. Water extract of *Morus alba* L. root bark had better efficacy for osteoarthritis treatment than aqueous extract of *Cornus officinalis* Siebold and Zucc fruits by increasing LBM and decreasing collagen degradation. Therefore, water extract of *Morus alba* L. root bark should be investigated as an alternative therapeutic agent for preventing osteoarthritis in testosterone-deficient elderly persons.

## Figures and Tables

**Table 1:** Contents of polyphenols and flavonoids in water extract of *Cornus officinalis* fruits (CFW) and *Morus alba* root bark (MBW).

Bioactive Components	CFW	MBW
Total polyphenol (mg GAE/g extract)	42.7 ± 1.1	18.2 ± 0.4
Total flavonoids (mg QE/g extract)	14.2 ± 0.7	2.3 ± 0.1
Gallic acid (μg/g extract)	0.45 ± 0.03	ND
Morroniside (μg/g extract)	11.0 ± 0.09	ND
Loganin (μg/g extract)	6.2 ± 0.04	ND
Kuwanon G (μg/g extract)	ND	2.2 ± 0.1
Morusin (μg/g extract)	ND	1.6 ± 0.1

QE, quercetin equivalent; GAE, gallic acid equivalent; ND, no detection.

**Table 2:** Metabolic parameters after 8- or 11-week treatments.

	ORX-CON (*n* = 10)	ORX-CFW (*n* = 10)	ORX-MBW (*n* = 10)	Non-ORX-CON (*n* = 10)
Bodyweight at 8th week (g)	387 ± 18 ^b^	382 ± 19 ^b^	385 ± 25 ^b^	479 ± 18 ^a^
Bodyweight gain at 8th week (g)	108 ± 13 ^b^	106 ± 13 ^b^	108 ± 12.9 ^b^	187 ± 21 ^a^
Bodyweight at 11th week	374 ± 17 ^b^	373 ± 16 ^b^	375 ± 19 ^b^	452 ± 17 ^a^
Bodyweight gain between 8 and 11 weeks (g)	−13 ± 2.1 ^b^	−9.0 ± 1.4 ^c^	10.1 ± 1.6 ^c^	−26.8 ± 2.1 ^a^
Food intake (g/day) at 11th week	17.3 ± 2.0	17.4 ± 2.2	18.4 ± 2.1	17.8 ± 1.5
Epididymal fat mass (g)	7.4 ± 1.2 ^a^	6.1 ± 1.1 ^c^	4.7 ± 0.9 ^c^	7.0 ± 1.5 ^ab^
Serum glucose (mg/dL) at 11th week	119 ± 14 ^a^	108 ± 11 ^ab^	105 ± 10 ^b^	110 ± 10 ^ab^
Serum insulin (ng/mL) at 11th week	2.2 ± 0.5 ^a^	1.5 ± 0.5 ^b^	1.4 ± 0.4 ^c^	1.8 ± 0.5 ^ab^
HOMA-IR at 11th week	7.8 ± 1.0 ^a^	4.8 ± 0.6 ^c^	4.4 ± 0.6 ^c^	5.9 ± 0.8 ^b^
Serum testosterone (ng/mL) at 11th week	0.88 ± 0.11 ^c^	1.23 ± 0.16 ^b^	1.27 ± 0.17 ^b^	1.87 ± 0.10 ^a^
Serum TNF-α (pg/mL)	68.4 ± 7.1 ^a^	56.5 ± 6.3 ^b^	50.1 ± 5.2 ^c^	59.3 ± 5.3 ^b^

The orchidectomized (ORX) rats were provided with a 43% fat diet containing (1) 0.5% dextrin (ORX-CON), (2) 0.5% water extract of *Cornus officinalis* fruits (ORX-CFW), or (3) 0.5% water extract of *Morus alba* root bark (ORX-MBW). Sham-operated rats had 0.5% dextrin in a 43 energy% fat diet (Non-ORX-CON) for 8 weeks. After 8 weeks of treatment, all rats had an injection of monoiodoacetate (MIA) into the left knee, and they continued the same diet for the additional 3 weeks. At the end of the experimental period, visceral fat (peri-uterine and retroperitoneum fat) mass and serum concentrations of testosterone and TNF-α, an inflammatory indicator, were measured. Values represent mean ± SD. ^a,b,c^ Means on the same row with different superscripts were significantly different by Tukey test at *p* < 0.05.

## Supplementary Materials

The following are available online at https://www.mdpi.com/1999-4923/12/12/1245/s1. Figure S1. HPLC chromatogram of water extracts of *Morus alba* L. root bark and *Cornus officinalis* Siebold and Zucc fruits. Figure S2. Serum glucose levels and area under the curve (AUC) during oral glucose tolerance test at 10th week. Figure S3. Changes of serum glucose levels during insulin tolerance test at 10th week.

## Abbreviations

MBW, water extract of *Morus alba* L. root bark; CFW, water extract of *Cornus officinalis* Siebold and Zucc fruit; ORX, orchiectomy; ORX-MBW, a group fed 0.5% MBW intake in testosterone deficient and osteoarthritis-induced rats; ORX-CFW, a group fed 0.5% CFW intake in testosterone-deficient and osteoarthritis-induced rats; ORX-CON, a group 0.5% dextrin intake in testosterone-deficient and osteoarthritis-induced rats; Non-ORX-CON, a group 0.5% dextrin intake in osteoarthritis-induced rats; TDS, testosterone-deficiency syndrome; OGTT, oral glucose tolerance test; IPITT, an intraperitoneal insulin tolerance test; HOMA-IR, homeostasis model assessment estimate of insulin resistance; MIA, monoiodoacetate; BMD, bone mineral density; LBM, lean body mass; MMP, matrix metalloproteinase; TNF-α, tumor necrosis factor-α; IL, interleukin; H-E, hematoxylin and eosin.
