# Supplementary material for: Aqueous Extracts of Morus alba Root Bark and Cornus officinalis Fruit Protect against Osteoarthritis Symptoms in Testosterone-Deficient and Osteoarthritis-Induced Rats
Source: Pharmaceutics. 2020 Dec 21;12(12):1245. doi: 10.3390/pharmaceutics12121245 (PMC7767081; doi:10.3390/pharmaceutics12121245)
Supplement: Supplementary file 1 [file pharmaceutics-12-01245-s001.pdf]

pharmaceutics

Article

Supplementary Materials: Aqueous Extracts of *Morus alba* Root Bark and *Cornus officinalis* Fruit Protect against Osteoarthritis Symptoms in Testosterone-Deficient and Osteoarthritis-Induced Rats

Sunmin Park, Bo Reum Moon, Ji Eun Kim, Hyun Joo Kim and Ting Zhang

A Kuwanon Morusin

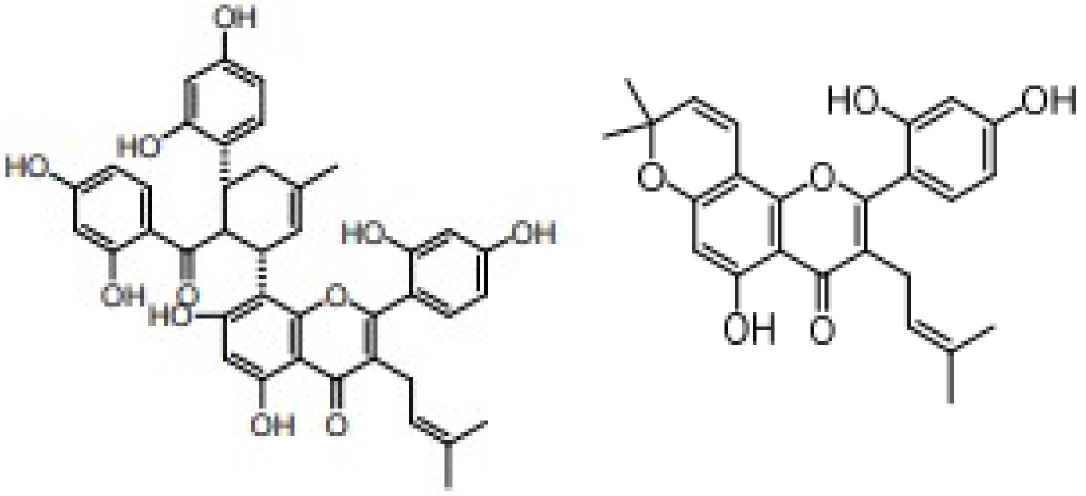

B

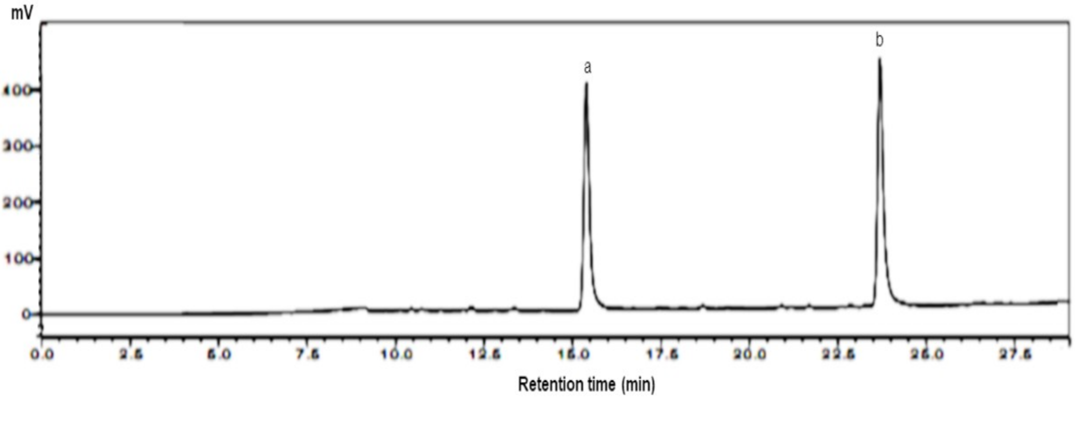

C

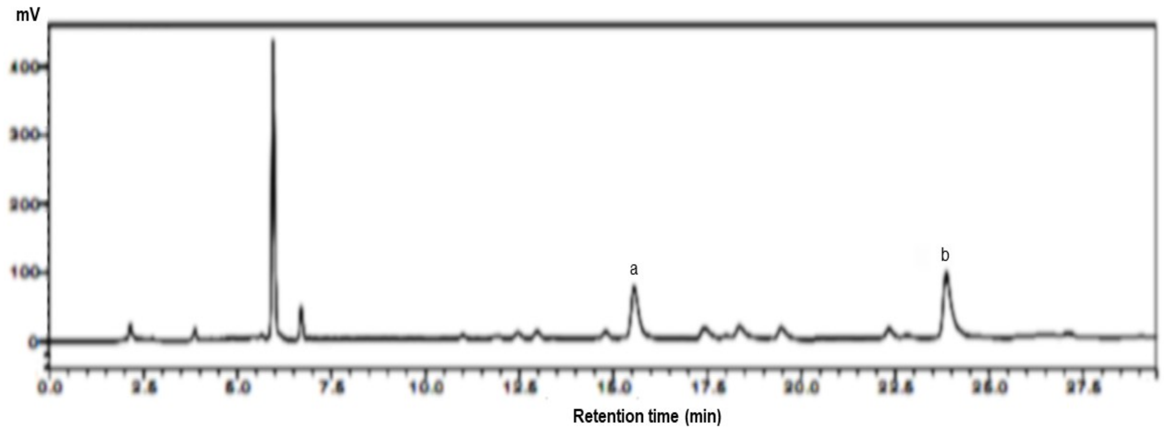

D Gallic acid Morroniside Loganin

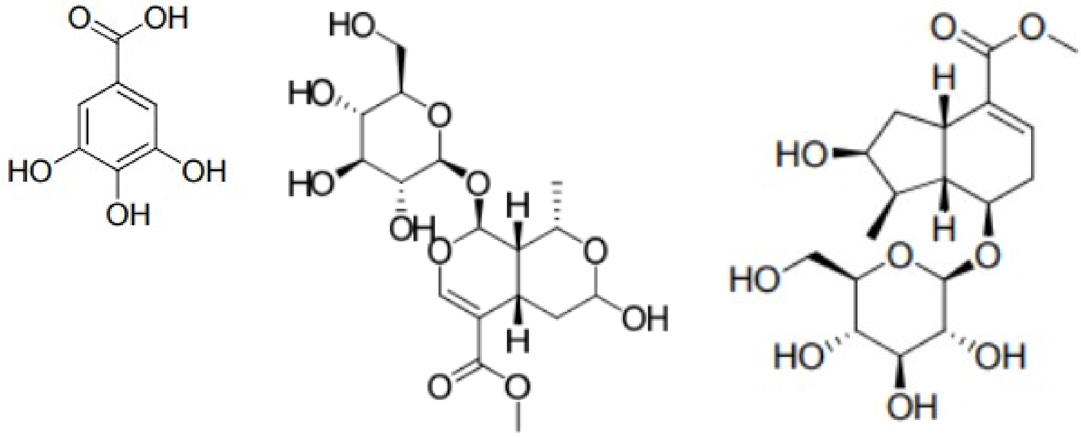

E

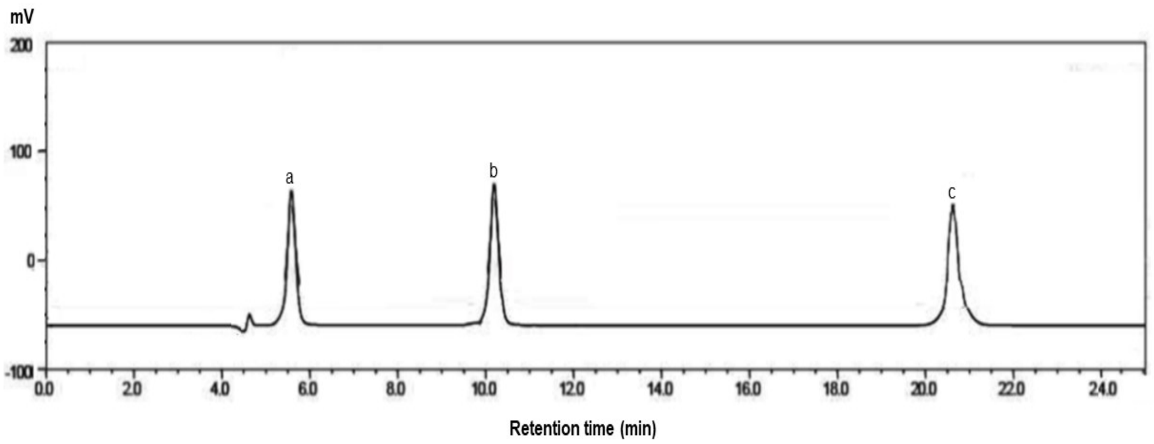

F

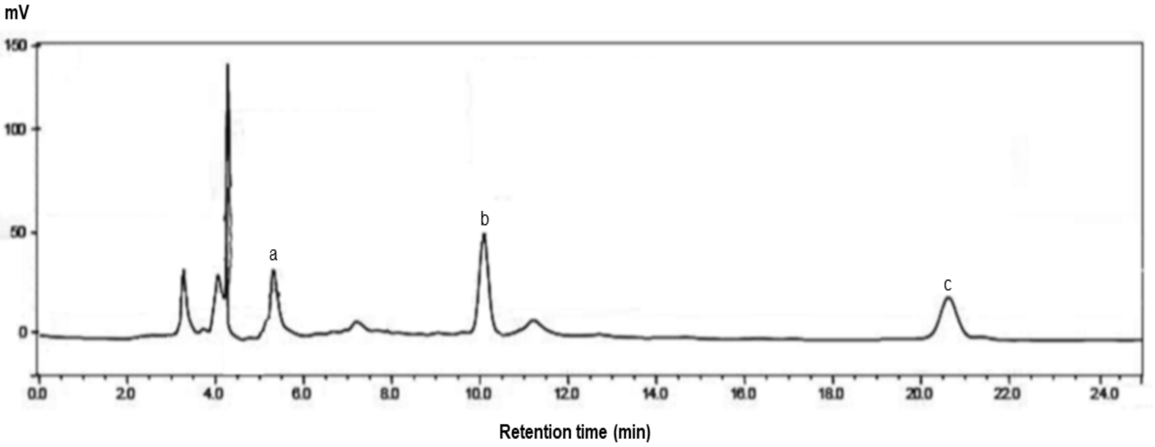

Figure 1. HPLC chromatogram of water extracts of *Morus alba* L. root bark and *Cornus officinalis* Siebold & Zucc fruits. (**A**) Chemical structure of kuwanon and morusin; (**B**) HPLC chromatogram of kuwanon and morusin standards; (**C**)HPLC chromatogram of water extracts of *Morus alba* L. root bark; (**D**) Chemical structure of gallic acid, morroniside, and loganin; (**E**) HPLC chromatogram of gallic acid, morroniside, and loganin; (**F**) HPLC chromatogram of *Cornus officinalis* Siebold & Zucc fruit.

A.

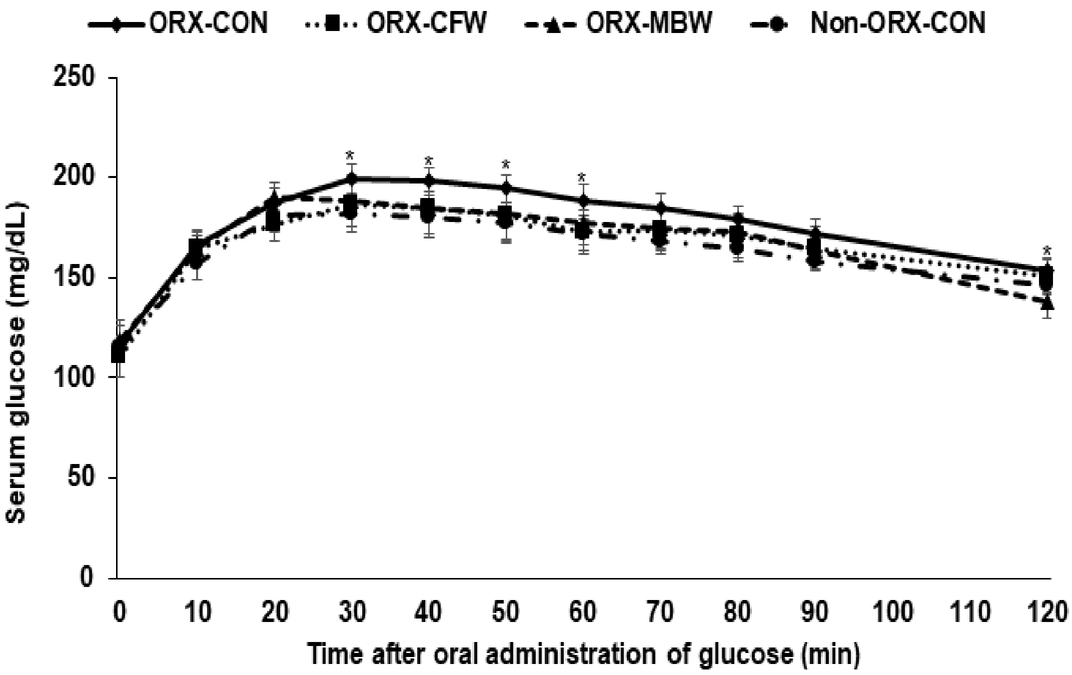

B.

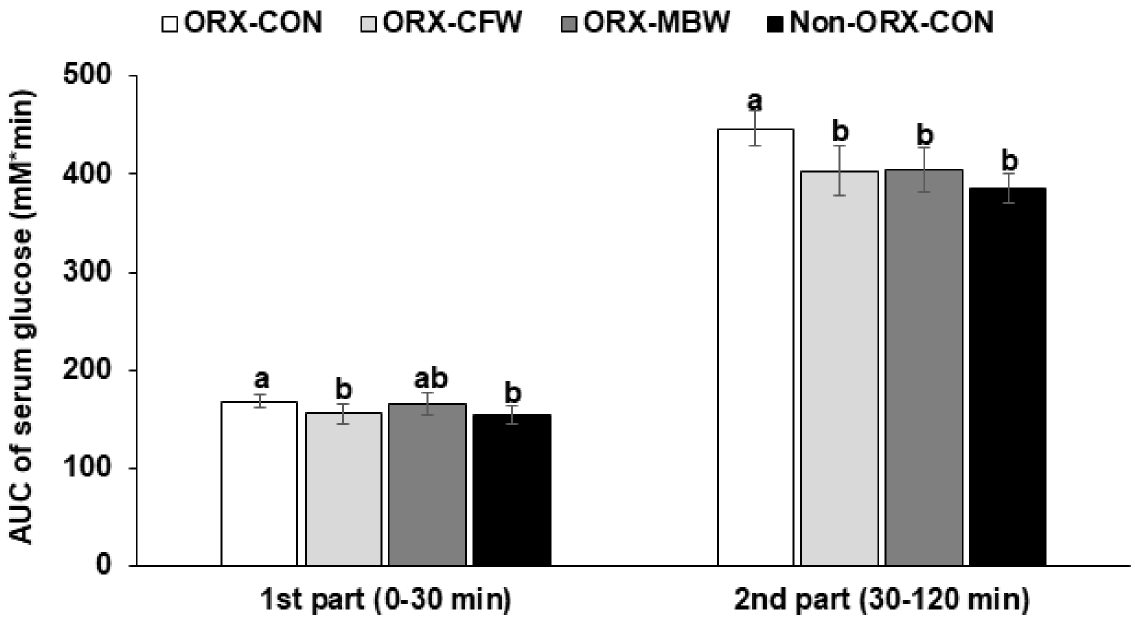

Figure 2. Serum glucose levels and area under the curve (AUC) during oral glucose tolerance test at 10th week. (**A**) Changes of serum glucose levels in 16-h fasted rats after oral challenge of 2 g glucose/kg body weight; (**B**) Area under the curve (AUC) of serum glucose calculated in the first (0–40 min) and second phases (40-120 min). Each bar or dot and error bar represented the mean ± SD, *n* = 10. Means without a common alphabet differ at P < 0.05. ORX-OA-CON, ORX male rats with 43% fat diet with 0.5% dextrin; ORX-OA-CFW, ORX male rats with 43% fat diet with 0.5% CFW; ORX-OA-MBW: ORX male rats with 43% fat diet with 0.5% MBW; Non-ORX-OA-CON, Sham male rats with 43% fat diet with 0.5% dextrin.

A

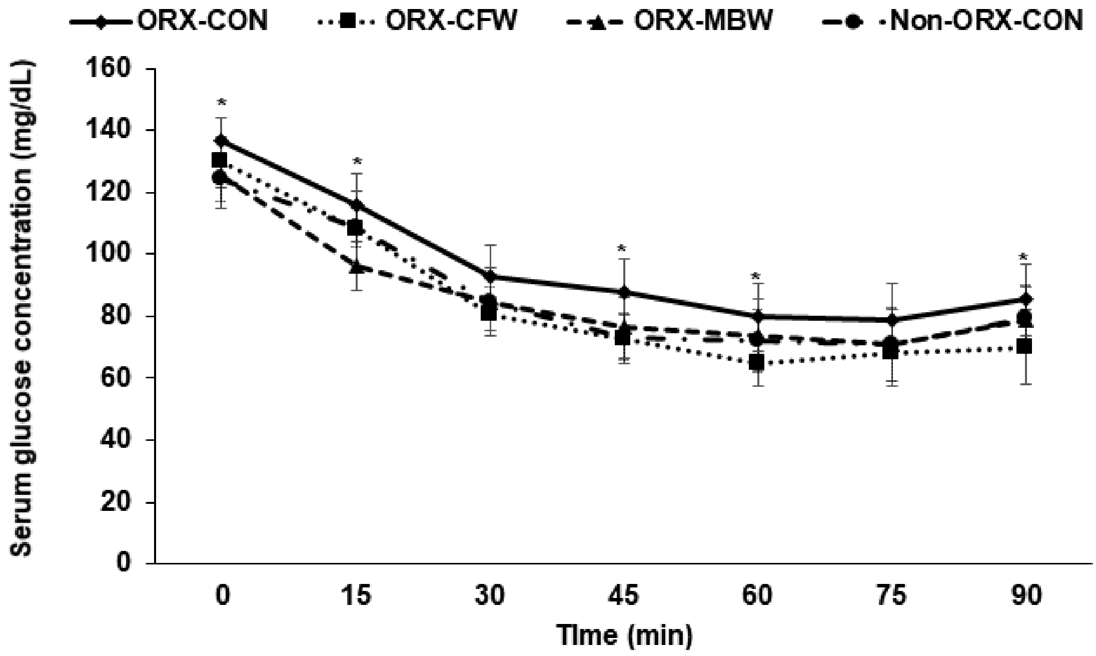

B

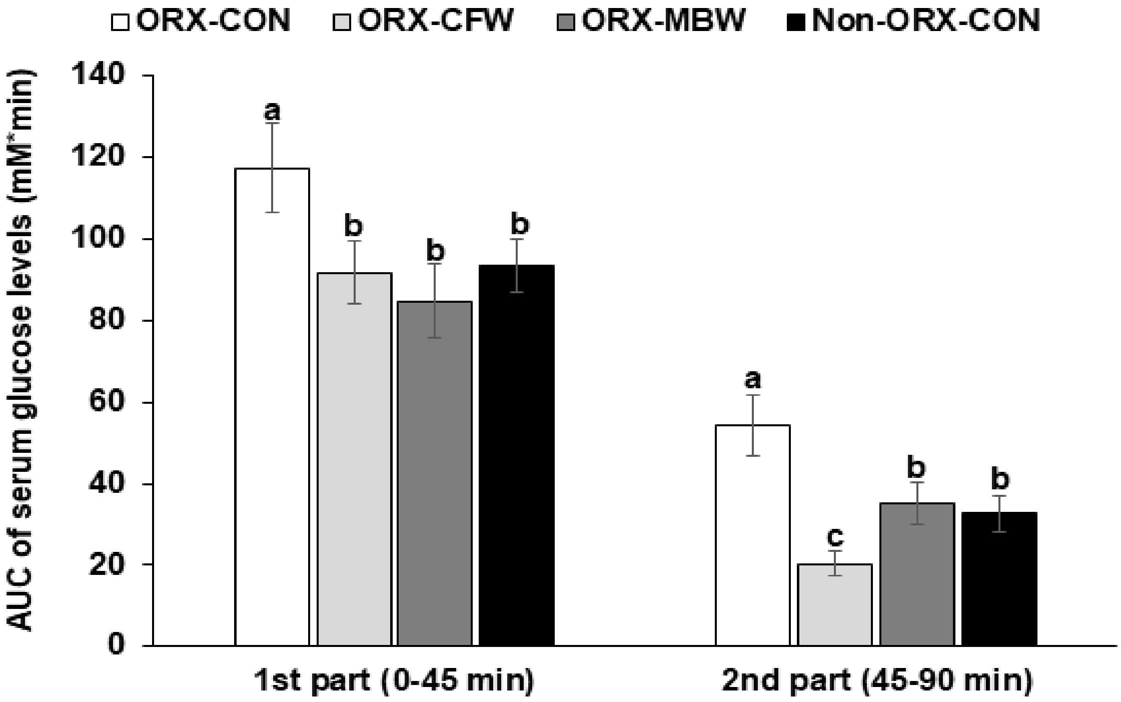

Figure 3. Changes of serum glucose levels during insulin tolerance test at 10th week; (**A**) Changes of serum glucose levels after 1 U insulin/kg body weight into subcutaneous injection after 6h food deprivation; (**B**) BArea under the curve (AUC) of serum glucose levels calculated in the first (0-30 min) and second phases (30–90 min). Each bar or dot and error bar represented the mean ± SD, $n = 10$. Means without a common alphabet differ at $P < 0.05$. ORX-OA-CON, ORX male rats with 43% fat diet with 0.5% dextrin; ORX-OA-CFW, ORX male rats with 43% fat diet with 0.5% CFW; ORX-OA-MBW: ORX male rats with 43% fat diet with 0.5% MBW; Non-ORX-OA-CON, Sham male rats with 43% fat diet with 0.5% dextrin.
